# Broiler Ascites Syndrome as a Potential Spontaneous Animal Model for Human Pulmonary Arterial Hypertension: A Narrative Review

**DOI:** 10.3390/life16050818

**Published:** 2026-05-14

**Authors:** Jie Zhang, Feihu Guan, Ye Tian, Yafen Song, Min Zhang, Xiaoyue Yang, Bing Zhang, Sifan Guo, Peng Ji, Chenghuai Yang, Min Yang, Qianyi Zhang

**Affiliations:** 1 China Institute of Veterinary Drug Control, Beijing 100081, China; lanmao_515@outlook.com (J.Z.); gfh9826@163.com (F.G.); ty18516952255@163.com (Y.T.); songyafen1@126.com (Y.S.); zmbooksea@sina.com (M.Z.); xiaoyue_yang2025@126.com (X.Y.); zhangbing06@163.com (B.Z.); 89gsf2008@sina.com (S.G.); ychenghuai@163.com (C.Y.); 2 College of Veterinary Medicine, Gansu Agricultural University, Lanzhou 730070, China; jip@gsau.edu.cn

**Keywords:** broiler ascites syndrome, pulmonary arterial hypertension, etiology, pathogenic mechanism, animal model

## Abstract

Pulmonary arterial hypertension (PAH), a major subtype of pulmonary hypertension (PH), is a significant human pulmonary disease whose pathogenic mechanisms remain incompletely understood. Conventional animal models for PAH induction often fail to fully replicate the entire disease progression, making it difficult to trace the initial causes, key pathogenic events, and early disease mechanisms. We propose an evidence-based hypothesis that Broiler Ascites Syndrome (BAS) exhibits high similarity to human pulmonary arterial hypertension in terms of pathogenic triggers and molecular pathology, suggesting its potential as a spontaneous animal model for PAH research. This review systematically summarizes the pathogenic mechanisms and disease-inducing factors of BAS, analyzing its pathobiological commonalities with PAH. We demonstrate shared features in pulmonary vascular remodeling mechanisms, including cellular dysfunction, tissue fibrosis, and immune dysregulation. Furthermore, consistent reactive mechanisms are observed across different types of PAH-related studies and corresponding poultry research. This collective evidence supports the feasibility of utilizing BAS as a spontaneous animal model for PAH investigation. By comparing the pathogenic mechanisms of BAS and PAH, this work provides novel insights for developing animal models in PAH research. If validated, this model could address limitations of existing models regarding hypoxia tolerance, and right heart remodeling.

## 1. Introduction

Broiler Ascites Syndrome (BAS), also known as Pulmonary Hypertension Syndrome (PHS), refers to a non-infectious group-occurring disease in broilers. It arises when a rapidly increasing metabolic level leads to systemic hypoxia, resulting in elevated pulmonary arterial pressure, subsequent right heart failure, increased central venous pressure, passive hepatic congestion, and ultimately the accumulation of serous fluid in body cavities [1,2]. The occurrence of this disease is influenced by multiple interacting factors, and its pathogenic mechanism is complex. The disease progression is often accompanied by pulmonary vascular remodeling and myocardial damage, which are considered irreversible hallmark features during its development. Currently, the incidence of BAS is on the rise. To effectively control its risk in farming practices, researchers have delved into the molecular pathological mechanisms, focusing on key processes such as the proliferation of pulmonary vascular smooth muscle cells, endothelial dysfunction, and the activation of the inflammation–fibrosis axis. By integrating predisposing factors like chronic hypoxia, genetic background, and metabolic stress, they have systematically analyzed the pathological basis of the disease, thereby deepening the understanding of the biological mechanisms of BAS. However, the etiology of BAS is complex, involving multifactorial interactions, and how these factors synergistically regulate and drive the occurrence and progression of the disease remains incompletely elucidated. Nevertheless, in current research progress, Pulmonary Arterial Hypertension (PAH) has shown significant similarities in pathological characteristics to BAS.

PAH is characterized by extensive loss of the pulmonary microcirculation, persistent vasoconstriction, and vascular remodeling, ultimately resulting in myocardial injury and progression to right heart failure. It is similarly a heterogeneous disease induced by multiple etiologies [3,4,5]. During disease progression, dysfunction of pulmonary vascular endothelial cells, abnormal proliferation of smooth muscle cells, adhesion and activation of immune cells, and the release of inflammatory factors collectively promote intimal thickening and loss of vascular compliance, thereby driving vascular remodeling [6,7]. As a relatively common cardiovascular disease in humans, it has a complex pathogenesis and numerous influencing factors, which pose certain difficulties in clinical research, and its pathogenic mechanisms have not been fully elucidated. Mechanistic studies have traditionally relied on mouse and rat models; rabbits, pigs, guinea-pigs and macaques have also been employed. These systems are technically demanding, costly, and often inadequately recapitulate the human phenotype [8]. In comparison to other animal models, chickens exhibit advantages such as short developmental cycles, high genetic diversity, and low cost. Furthermore, core immunological concepts, including the biology of B/T lymphocytes, mechanisms of immune tolerance induction, and graft-versus-host disease, originally emerged from research on chickens. The chicken immune system demonstrates high conservation with humans in terms of developmental processes, composition of cellular subsets, and response patterns. Additionally, homologous genes account for an average of 75.3% of the chicken genome compared to humans, establishing chickens as a classic non-mammalian vertebrate model for deciphering fundamental immunological principles and modeling human immune-related diseases.

**Figure 1:**
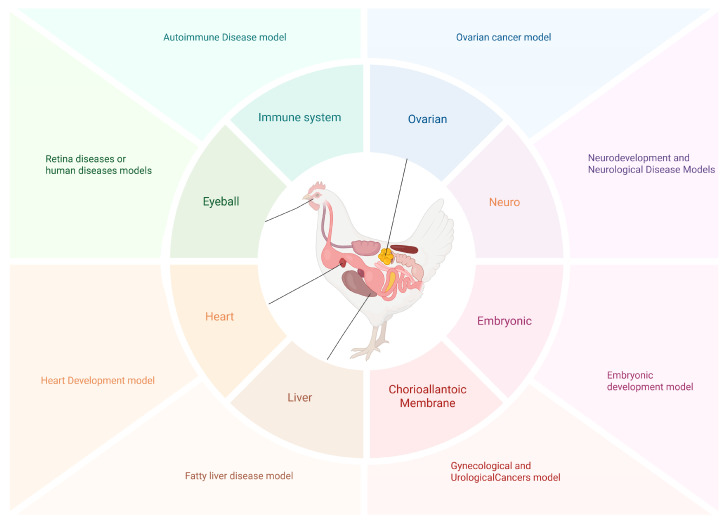
Application of Different Tissues and Organs of Chickens in Animal Models. The heart is used as a model for early cardiac development. The eye serves as an important research model for hereditary and non-hereditary ocular diseases such as pathological myopia, retinal detachment and ocular tumors. The immune system acts as a model for spontaneously occurring autoimmune diseases. The ovary is applied as a disease model of human ovarian cancer. The liver serves as a disease model of human fatty liver. The embryo is used as a model for embryonic development. The chick chorioallantoic membrane (CAM) can be used as an in vitro tumor research model. Created in BioRender. Zhang, J. (2026) https://BioRender.com/rfibvfj.

In human disease research, the selection of animal models requires comprehensive consideration of multiple factors, including similarities to human anatomical, physiological, or genetic characteristics, as well as research cycle, cost, and ethical concerns [9]. Among various animal models, chickens are widely used in multiple research fields such as developmental biology, virology, immunology, oncology, epigenetic regulation of gene expression, conservation biology, and domestication genomics due to their rapid development, low maintenance costs, high genetic diversity, and unique physiological characteristics. Specifically, chickens and chicken embryos have been extensively utilized in constructing disease models for ophthalmology, cancer, gynecological and urological cancers, abnormal embryonic development, influenza, spontaneous autoimmune diseases, fatty liver disease, and hyperosteogeny, achieving significant outcomes (Figure 1). For instance, the ocular structure and physiological characteristics of chickens exhibit a high degree of similarity to those of humans, making them valuable models for studying both genetic and non-genetic ocular diseases such as pathological myopia, retinal detachment, and ocular tumors [9,10,11]. Additionally, the chorioallantoic membrane of chickens, as an easily accessible extraembryonic tissue with a rich vascular system, enables in vitro tumor studies. Moreover, given the high morphological and histopathological similarity between chicken ovarian cancer and human ovarian cancer, chicken models offer greater convenience, cost-effectiveness, and practical utility compared to mouse models in the study of gynecological and urological tumors [12,13,14].

Compared to experimentally induced models, spontaneous animal models can more accurately reflect the natural pathological processes of diseases. Autoimmune diseases often manifest clinical symptoms only after the compensatory capacity of organs or systems is exhausted, making it difficult to trace their initial etiology, pathogenic events, and early pathogenesis. Given the high similarity between the chicken immune system and human immune responses in multiple aspects, spontaneously occurring chicken models of autoimmune thyroiditis, scleroderma-like disease, and vitiligo-like syndrome have been utilized to study and analyze related human diseases [15,16,17], serving as important tools for elucidating early pathological responses and genetic factors. In cardiovascular disease research, numerous spontaneous animal models have been employed, with rats and mice being the most commonly used. However, no studies have yet utilized broilers as spontaneous animal models for cardiovascular diseases. Notably, chickens represent the earliest model organism for studying cardiac development, with cardiovascular developmental processes sharing similarities with humans [18]. Furthermore, broiler ascites syndrome demonstrates pathological processes identical to human pulmonary arterial hypertension in mechanistic studies, as well as shared disease predisposing factors such as nutritional imbalances, hypoxia, and low temperatures. Therefore, this review will focus on elucidating the pathogenesis and predisposing factors of broiler ascites syndrome, further explore its pathological commonalities with human pulmonary arterial hypertension, and clarify the feasibility of utilizing chickens affected by broiler ascites syndrome as spontaneous animal models for human pulmonary arterial hypertension.

## 2. Mechanism of Pulmonary Arterial Remodeling

**Figure 2:**
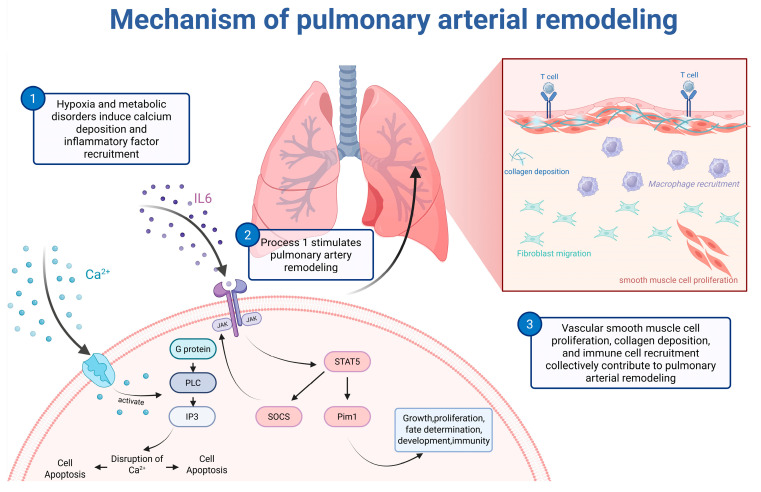
Mechanism of pulmonary arterial remodeling. Ca^2+^ upregulates the expression of the intracellular calcium-sensing receptor (CaSR), activating the downstream G protein-PLC-IP3 pathway. This disrupts Ca^2+^ homeostasis, leading to cell proliferation and apoptosis. Inflammatory cytokines activate the Jak-STAT pathway, promoting the expression of genes such as Pim1 and SOCS3. This triggers a series of cellular activities, including cell proliferation, apoptosis, and immune responses. In normal pulmonary arteries, disruptions in ion homeostasis and dysregulated immune responses trigger two main pathological processes: the abnormal proliferation of fibroblasts and smooth muscle cells with excessive secretion of extracellular matrix components such as collagen, and the adhesion of immune cells accompanied by the release of inflammatory factors. These processes collectively drive vascular fibrosis, leading to the remodeling of the pulmonary artery. Created in BioRender. Zhang, J. (2026) https://BioRender.com/8mxa96m.

Pulmonary arterial remodeling represents a central step in the pathogenesis of BAS. This pathological process is highly complex, involving interactions among multiple cell types, active mediators, signaling pathways, and cytokines. It ultimately leads to a series of pathological changes, including vascular wall thickening, inflammatory responses, apoptosis, and abnormal proliferation and phenotypic transformation of smooth muscle cells. These changes collectively contribute to increased pulmonary vascular resistance and elevated pulmonary arterial pressure. As the primary constituent cells of the pulmonary arterial media, smooth muscle cells have been a major focus. Studies utilizing cold-induced broiler ascites models have observed a correlation between the level of smooth muscle cell proliferation and changes in the relative medial thickness and relative wall area of pulmonary arteries, suggesting that abnormal smooth muscle cell proliferation is a primary driver of pulmonary arterial remodeling [19]. Based on this observation, subsequent research has further explored the molecular mechanisms underlying this abnormal proliferation. Evidence indicates that CaCl_2_ can significantly upregulate the expression of the calcium-sensing receptor (CaSR), increase Ca^2+^ deposition, and promote the abnormal proliferation of pulmonary arterial smooth muscle cells under hypoxic conditions via activation of the G protein-PLC-IP3 pathway [20]. Furthermore, Fei et al. [21] conducted transcriptomic sequencing on pulmonary arteries from both normal chickens and ascitic chickens at day 29 of induced disease. Their screening identified involvement of the ribosome, Jak-STAT, and NOD-like receptor signaling pathways, and revealed that key genes such as *STAT5*, *Pim1*, and *SOCS3*, along with cytokines including IL-6, IL-8, IL-1β, IL-18, and MIP-1β, participate in mediating inflammatory responses that drive smooth muscle cell proliferation, thereby promoting pulmonary arterial remodeling (Figure 2). To delve deeper into the core regulatory genes involved in this remodeling, further filtering of predicted genes was performed. Among these, hypoxia-inducible factor-1α (HIF-1α) was repeatedly identified as a significant target gene and also plays a role in microRNA dysregulation observed in ascitic chickens [22]. As a master regulator of the hypoxic response, HIF-1α promotes the proliferation and apoptosis of pulmonary arterial smooth muscle cells, endothelial cells, and fibroblasts, and modulates gene expression. It also facilitates the growth of fibrous tissue and collagen deposition within the pulmonary arterial adventitia, thereby exacerbating the thickening and remodeling of the vessel’s three-layer structure [23,24]. While chronic pulmonary arterial remodeling and hypertension can lead to right heart failure or portal vein obstruction, ultimately leading to ascites formation, the precise causal relationship between these two pathological phenomena has not been clearly elucidated by research. Moreover, given that BAS is fundamentally an energy metabolism-related disease, cardiac energy metabolism is likely to play a critical role in its progression.

## 3. Mechanisms of Myocardial Injury

**Figure 3:**
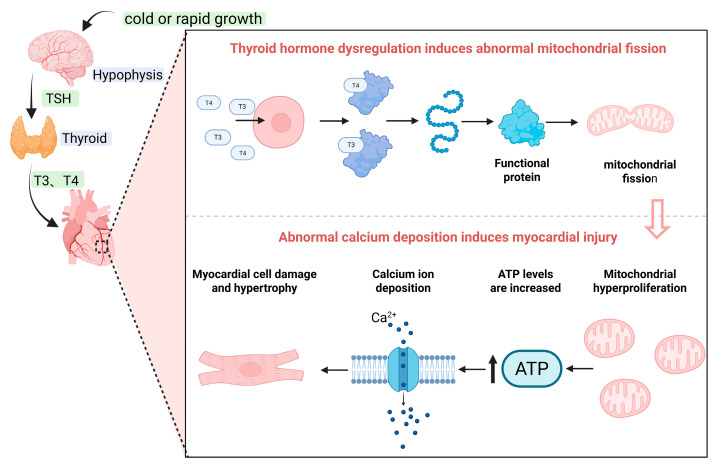
Mechanisms of myocardial injury. Cold exposure and rapid growth stimulate the hypothalamus to promote the release of thyroid-stimulating hormone (TSH) from the adenohypophysis, which in turn induces the thyroid gland to secrete thyroid hormones (triiodothyronine, T3, and thyroxine, T4). Upon entering cardiomyocytes, T3 and T4 activate gene transcription, leading to the regulation of mitochondrial fission and a consequent increase in mitochondrial biogenesis. The resulting elevated ATP production enhances calcium influx through calcium channels. When sustained over time, this persistent calcium influx promotes intracellular calcium deposition, ultimately causing cardiomyocyte injury and hypertrophy. Created in BioRender. Zhang, J. (2026) https://BioRender.com/5itf0nv.

The heart, as one of the most critical target organs in BAS, demonstrates significant pathological alterations during disease progression. These include dysregulation of myofibril-structured cardiomyocytes, substantial deposition of blood cells within the intermyofibrillar vasculature, and concomitant energy metabolism disturbances, collectively leading to myocardial injury and subsequent right heart failure [25]. Research indicates that during the rapid growth phase of broilers, substantial secretion of thyroid hormones enhances metabolic efficiency and increases oxygen consumption. When oxygen demand exceeds the respiratory system’s capacity, the risk of BAS is significantly elevated [26]. Furthermore, persistently high levels of thyroid hormone secretion can induce hyperthyroidism, characterized by an upregulation of β-adrenergic receptor density in the myocardium, alongside enhanced binding capacity and affinity for thyroid hormones. This dysregulation promotes cardiomyocyte apoptosis [27] and can lead to myocardial injury. Concurrently, it may increase Ca^2+^ influx and the efficiency of calcium release from the sarcoplasmic reticulum (Figure 3). Intracellular Ca^2+^ flux plays an indispensable role in numerous fundamental cellular activities. Calmodulin, regulated by Ca^2+^, is a key modulator mediating both growth and function in cardiomyocytes. Cardiac troponin T, a crucial early diagnostic biomarker for human myocardial injury, is also significantly upregulated in ascitic broilers, suggesting its potential involvement in mediating myocardial damage and its utility as a potential early marker in BAS [28]. Moreover, during excitation-contraction coupling in the myocardium, precise regulation of Ca^2+^ is essential for maintaining contractile function. In human heart failure, interventions targeting disordered calcium cycling are employed to improve systolic dysfunction. In ascitic broilers, extensive Ca^2+^ deposition is observed in the right ventricle, alongside a near absence of Ca^2+^-ATPase reaction products on the membranes of the myocardial sarcoplasmic reticulum and mitochondria. This deficiency likely constitutes a critical pathological basis for right ventricular diastolic dysfunction [29]. However, the precise molecular mechanisms underlying the reduced Ca^2+^-ATPase activity and abnormal calcium cycling regulation remain incompletely elucidated. In mammals, myocardial injury often downregulates TRPC1 expression, thereby affecting the function of the canonical transient receptor potential cation channels and modulating intracellular Ca^2+^ levels through this pathway. In contrast, avian species appear to exhibit a different response, potentially relying on other, yet unidentified, pathways to regulate intracellular Ca^2+^ homeostasis [30]. To further analyze the molecular mechanisms of metabolic disturbance in the myocardium, Karim Hasanpur et al. [31] performed transcriptomic sequencing on right ventricular tissue collected on day 21 of induced disease onset. Screening identified differentially expressed genes primarily enriched in pathways related to bacterial defense response, biological adhesion, cell adhesion, heterologous cell killing, cell division, PPAR signaling pathway, fatty acid metabolism, and adipocytokine signaling. Interestingly, this myocardial transcriptomic data revealed that the activation timing of these metabolism-related pathways precedes the initiation of the inflammatory cytokine cascade associated with pulmonary arterial remodeling described previously. This suggests that metabolic dysregulation may be the primary molecular event preceding the onset of ascites syndrome, while pulmonary arterial remodeling represents a downstream, secondary structural adaptation.

## 4. Etiology of BAS

### 4.1. Genetic Factors

Studies have demonstrated that traits associated with BAS exhibit high heritability in broiler populations. During early post-hatch development, arterial hemoglobin oxygen saturation shows a significant genetic correlation with susceptibility to BAS. Together with hematocrit levels, it plays a critical role in determining the blood’s capacity for oxygen transport. In affected ascitic broilers, reduced hematocrit has been positively correlated with body weight [32,33]. A. Pakdel’s [34] research on the relationship between growth efficiency and ascites susceptibility revealed that each 130 g increase in body weight gain is associated with heightened disease susceptibility. Consequently, the ability to maintain adequate oxygen supply during periods of rapid weight gain significantly affects the incidence of BAS. In long-term genetic selection programs, emphasis has largely focused on enhancing growth rate and muscle yield, while cardiopulmonary functional capacity has been comparatively neglected. This imbalance has resulted in a physiological mismatch: accelerated growth elevates metabolic demand and oxygen consumption, but the cardiopulmonary system fails to adapt accordingly, leading to the development of BAS [35]. A comparable condition is observed in preterm human infants, in whom impaired alveolar and vascular development can lead to pediatric pulmonary hypertension, frequently accompanied by metabolic disturbances [36].

Mitochondria, the primary energy producers of the cell, are fundamentally linked to metabolic processes in broilers. Possessing a short genome that is inherited maternally, mitochondria are regulated in their abundance and distribution by specific proteins such as Drp1, MTFP1, Opa1, and AMP-activated protein kinase (AMPK). These proteins are critical for governing cellular energy metabolism and maintaining cellular function. Mitochondrial dysfunction and locus-specific defects in the electron transport chain may contribute to the development of BAS [37,38]. In this context, Khaloud Al-Zahrani et al. [39] demonstrated a strong, tissue-specific correlation between mitochondrial DNA (mtDNA) copy number and the ascites phenotype. Their research further proposed the mtDNA-to-nuclear DNA (nucDNA) ratio as a potential predictive biomarker for breeding programs designed to reduce the incidence of ascites. Additionally, the capacity for mitochondrial biogenesis has been shown to differ significantly among various broiler lines and genotypes [40].

Furthermore, to mitigate the high incidence of BAS associated with the selection for fast-growing broilers, genetic breeding strategies aimed at developing ascites-resistant lines are being pursued. Genome-wide SNP analyses have identified several candidate genes potentially involved in this trait (including *MC4R*, *MEF2c*, *CDH6*, *CPQ*, *LRRTM4*, *AGTR1*, and *UTS2D*) [41,42,43,44,45]. Polymorphisms in *MC4R*, a gene linked to obesity and cardiovascular diseases in other species, may influence cardiovascular development through interactions with dietary factors [46]. *CDH6*, which encodes a surface receptor protein on platelets [47], regulates platelet aggregation [48], inhibition of CDH6 expression reduces thrombus formation [49], Additionally, glycyl-tRNA synthetase has been shown to promote vascular endothelial inflammation and angiogenesis via *CDH6* [50], This mechanism is relevant to BAS, which involves vascular inflammation during pulmonary arterial remodeling. SNP-specific analysis has revealed a strong association between *CPQ* and BAS. This gene is linked to hypertension and blood pressure regulation in humans, and notably, chickens develop plexogenic arteriopathy similar to certain forms of human pulmonary hypertension [1], In quantitative trait locus analyses, *LRRTM4* was found to interact with *CPQ*. Specifically, male-specific epistasis was observed between *LRRTM4* heterozygotes and non-reference homozygotes in intron 6 of *CPQ*, resulting in greater BAS resistance in male chickens [44]. *AGTR1* (Angiotensin II Receptor Type 1) and *UTS2D* (Urotensin II-Related Peptide), both associated with hypertension and hypoxic responses in mammals, are located in genomic regions linked to ascites susceptibility and exhibit significant sex-specific effects in chickens [43].

Transcriptomic analyses have identified several genes undergoing potential adaptive mutations in the context of BAS, including *ALDH7A1*, *IRG1*, *GGT5*, *IGSF1*, *DHX58*, *USP36*, *TREML2*, *SPAG1*, *CD34*, and *PLEKHA7*. Among these, *ALDH7A1* plays a critical role in pulmonary arterial remodeling and vasoconstriction [51]. *IRG1* exerts a key protective effect by suppressing inflammation and preventing cardiac dysfunction under conditions of ischemic or toxic injury [52], It also protects against cholesterol-induced inflammation and atherosclerosis via the itaconate axis [53]. *IGSF1* is involved in the central regulation of thyroid hormones [54], and its mutation is a common genetic cause of thyroxine deficiency [55], which can adversely affect the cardiovascular system. Dysregulated thyroid hormone secretion is likewise implicated in BAS pathogenesis. *DHX58* shows a potential association with coronary artery disease, linked to reduced risk [56]. Structural variations in *USP36* are significantly associated with coronary artery disease and influence multiple cardiometabolic traits [57]. Specific genetic variants of *TREML2* correlate with hypertension in males [58], Stem cells expressing CD34 are major contributors to cardiac remodeling following myocardial injury from ischemia or reperfusion and can induce post-injury vascular development [59]. *PLEKHA7* may influence blood pressure regulation and cardiovascular function through its effects on the vasculature, with certain mutations attenuating salt-sensitive hypertension in rats [60]. Genomic screening in ascitic broilers has thus revealed numerous candidate genes associated with BAS. These genes appear to undergo adaptive mutations during selection, and notably, many are also associated with human cardiovascular diseases.

### 4.2. Environmental Factors

BAS was initially identified in high-altitude regions. At elevated altitudes, reduced atmospheric pressure leads to a decline in oxygen partial pressure, resulting in systemic hypoxia and diminished respiratory efficiency in broilers. Due to intensive genetic selection, modern broilers are predisposed to various metabolic disorders, with hypoxia serving as a primary trigger for these dysregulations [61]. Although BAS manifests predominantly during the rapid growth phase, evidence suggests its etiology may originate as early as the embryonic stage [62]. In broiler lines susceptible to ascites, thyroid hormone levels during late incubation are significantly lower than in normal birds. These hormones play a vital role in synthesizing pulmonary surfactant and are essential for proper respiratory system development. Consequently, their deficiency leads to delayed maturation of the respiratory system, prolonged hatching duration, and reduced oxygen availability within the air cell, collectively establishing a hypoxic internal environment that increases BAS susceptibility [63]. Furthermore, chronic embryonic hypoxia induced by low oxygen partial pressure during incubation can cause abnormal embryonic structure and cardiovascular dysfunction [64], This may explain the higher BAS-associated mortality observed at high altitudes compared to lowlands. Conversely, elevating carbon dioxide concentrations in the incubator during the late incubation phase can induce adaptive responses in chicken embryos, subsequently reducing the incidence of BAS during the growth period [62,65]. Therefore, the mechanism by which the high-altitude environment promotes BAS is more complex than simply reducing oxygen levels.

Low temperature is also a major inducer of this disease. It increases the secretion of thyroid hormones and the demand for oxygen in broilers, leading to increased cardiac output and elevated pulmonary arterial pressure [66], Under low-temperature conditions, the ratio of right ventricular weight to total ventricular weight exhibits a strong positive genetic correlation with ascites mortality [67]. During the brooding process, reduced brooding temperature increases mortality from ascites during growth [68], In the final stage of broiler embryogenesis, repeated brief acute cold exposure can induce chicks to produce more heat, enhancing their ability to resist cold throughout the life cycle and reducing the incidence of BAS [69]. Therefore, the choice of incubation temperature is crucial for the occurrence of BAS during broiler growth. After hatching, during the rapid growth period (3–5 weeks of age), low temperature can increase the expression of thyroid hormones and their receptor gene (*THRα*) in broilers, enhancing cellular metabolism and increasing cellular oxygen consumption and carbon dioxide production [70], Furthermore, the expression of many genes related to contractile elements (*MHCα*, *RYR2*, and *SERCA2*) increases, leading to adaptive right ventricular hypertrophy, impaired cardiac systolic function, and the occurrence of ascites [71]. Subsequently, the expression level of the mitochondrial isoform of phosphoenolpyruvate carboxykinase (M-PEPCK), involved in the gluconeogenesis process, increases, and hepatic oxidative damage is significantly aggravated [72]. The low-temperature environment induces metabolic disorders during the development of BAS. Two biological pathways—tryptophan biosynthesis metabolism and glycerophospholipid metabolism—may contribute to the induction of BAS. Moreover, potential metabolic biomarkers in glycerophospholipid metabolism may have a key regulatory function in BAS induced by cold stress [73,74].

### 4.3. Nutritional Factors

Nutrition is not a primary factor in the onset of BAS, but rather acts in concert with environmental and genetic factors to promote its development. In production settings, to maximize profitability, broilers are often fed high-nutrient-density diets to accelerate growth rate. However, this rapid growth can overwhelm the physiological capacity of internal organs due to the high metabolic demands, leading to the occurrence of BAS. Early management practices utilized restricted feeding to mitigate the incidence of the disease, though this approach adversely affected production efficiency. To reduce ascites incidence without compromising broiler productivity, the use of feed additives has been explored. Studies have shown that supplementing conventional diets with L-arginine increases the production of nitric oxide (NO), a vasodilatory molecule, thereby inducing pulmonary vasodilation. This reduces pulmonary arterial pressure and pulmonary vascular resistance, decreases right ventricular hypertrophy, and lowers the incidence of ascites. Similar beneficial effects have been observed following in ovo injection of arginine [75,76,77,78]. However, supplementing L-arginine in conventional diets beyond NRC-recommended levels conflicts with feed additive standards. Consequently, research has investigated the addition of vitamin E to counteract the loss of NO bioavailability caused by oxidative radicals. Nevertheless, this approach did not significantly improve pulmonary vasodilation or reduce BAS mortality [79,80]. In contrast, subsequent studies reported opposing results, indicating that dietary vitamin E supplementation significantly increased daily weight gain in broilers, downregulated angiotensin-converting enzyme expression in lung tissue, and reduced the incidence of BAS [81], Furthermore, subcutaneous implantation of vitamin E attenuated lipid peroxidation processes and effectively mitigated PHS-induced mortality in broilers [82]. These discrepant outcomes may be attributed to differences in supplementation methods and husbandry practices. Additionally, additives such as zinc sulfate, selenium, and N, N-dimethylglycine have been shown to ameliorate BAS induced by environmental hypoxia, cold stress, and high metabolic efficiency. Their beneficial effects are mediated through the regulation of free radical production, reduction in hepatic lipid peroxidation, and improvement of feed conversion ratio [83,84,85].

An imbalance in dietary composition can also lead to the occurrence of ascites. For instance, excessive supplementation of sodium bicarbonate can reduce erythrocyte deformability and increase blood volume, potentially triggering right ventricular failure and ascites [86,87], however, supplementation at lower levels may help prevent its occurrence. While salt is an essential dietary component, its addition to drinking water at various concentrations can aggravate BAS and cause renal damage. Furthermore, excessive intake upregulates *HIF-1α* expression in cardiac and pulmonary tissues, thereby inducing BAS [88,89]. Dietary protein level is another significant factor. In an experiment conducted by Behrooj et al. [90] broilers raised at high altitude (2100 m) were fed diets with varying protein levels. Results demonstrated that birds receiving low-protein diets experienced a significant increase in BAS-associated mortality.

## 5. Comparability of PAH and BAS

Pulmonary Hypertension (PH) encompasses various classifications. Based on similar pathophysiological mechanisms, clinical presentations, hemodynamic characteristics, and therapeutic management, the World Health Organization (WHO) has clinically categorized PH into five major groups: “Pulmonary Arterial Hypertension (PAH)”, “PH due to left heart disease”, “PH due to lung diseases and/or hypoxia”, “PH due to pulmonary artery obstructions”, and “PH with unclear and/or multifactorial mechanisms”. According to international guidelines, the diagnosis of PH requires a mean pulmonary arterial pressure (mPAP) ≥ 20 mmHg at rest as measured by right heart catheterization [91]. The first major subgroup, PAH, has stricter hemodynamic criteria, specifically referring to pre-capillary pulmonary hypertension resulting from intrinsic pulmonary arterial pathology. It is characterized by increased pulmonary vascular resistance due to “pulmonary vasoconstriction, endothelial dysfunction, smooth muscle proliferation, and fibrosis”, ultimately leading to right heart failure. PAH can be further classified into “Idiopathic PAH”, “Heritable PAH”, “Drug- and toxin-induced PAH”, “PAH associated with specific conditions”, “PAH with overt features of venous/capillaries (PVOD/PCH) involvement”, and “Persistent PH of the newborn syndrome (PPHN)” [92,93]. The following sections compare the pathogenic mechanisms and distinct classifications of PAH.

### 5.1. Pathogenic Mechanism

PAH is a heterogeneous disease with diverse etiologies and complex pathophysiological mechanisms, including endothelial metabolic reprogramming, sex hormone signaling, cellular dysfunction, vascular fibrosis, and right ventricular–pulmonary arterial (RV–PA) coupling. Recent evidence highlights subtype-specific molecular features. In hypoxia-associated PAH, the HIF-1, TNF, and IL-17 pathways are significantly enriched, accompanied by marked expansion of pulmonary arterial smooth muscle cells (SMCs). A hypoxia-synthetic SMC subpopulation has been identified as a key driver of vascular remodeling, establishing SMCs as central effectors in hypoxia-induced disease progression. In contrast, PAH with lower hypoxic signatures is more closely associated with metabolic and physiological processes [94]. Although the TGF-β/SMAD pathway has been widely recognized as a driver of vascular fibrosis, it fails to fully explain the observed heterogeneity. Alternative mechanisms have emerged, including aldosterone-induced endothelial secretion of NEDD9-enriched extracellular vesicles. These vesicles are internalized by SMCs and fibroblasts, leading to upregulation of NEDD9, increased collagen I/III production, and pan-vascular wall fibrosis, independent of TGF-β signaling [95]. Hypoxia-inducible factor-2α (*HIF-2α*) further contributes to disease progression by regulating the lncRNA KMT2E-AS1, stabilizing KMT2E protein and promoting H3K4me3 histone modification. This epigenetic axis drives endothelial metabolic reprogramming toward glycolysis, as well as aberrant proliferation and vascular remodeling [96]. Sex hormone signaling also plays a dual role in PAH. In BMPR2 mutant mouse models, inhibition of estrogen synthesis prevents and reverses PAH, accompanied by improvements in metabolic dysfunction, including insulin resistance, impaired lipid oxidation, ceramide accumulation, and downregulation of the PPAR-γ/CD36 axis, as well as reduced mitochondrial superoxide production [97]. Paradoxically, female patients with PAH exhibit superior right ventricular function and better prognosis than males, a phenomenon termed the “estrogen paradox” [98]. Right ventricular function is a critical determinant of clinical outcomes in PAH. RV–PA coupling has been identified as a strong prognostic indicator (HR = 0.19), closely associated with mortality risk [99]. However, Ees/Ea is insufficient to identify early irreversible right ventricular failure and shows improvement only in late-stage “super-responders”. In contrast, right ventricular diastolic function appears to be a more reliable predictor of therapeutic response in advanced disease [100]. Collectively, these findings underscore the mechanistic heterogeneity of PAH, with distinct regulatory pathways operating across different disease subtypes.

Although BAS and PAH are diseases that can cause cardiovascular disease in different species, they exhibit significant similarities in their pathological progression and disease etiology. The pathological progression is characterized by pulmonary arterial remodeling, leading to increased pulmonary arterial pressure, right ventricular myocardial injury, and progression to right heart failure. Both diseases share common pathological features, including pulmonary vascular remodeling, inflammation-mediated tissue fibrosis, cellular dysfunction, and right heart failure. During the process of pulmonary arterial remodeling, the two diseases demonstrate consistent cellular dynamics. Endothelial cells undergo endothelial-mesenchymal transition, fibroblasts differentiate into myofibroblasts, and smooth muscle cells switch from a contractile to a synthetic phenotype. All three cell types exhibit abnormal proliferation and secrete excessive extracellular matrix components, such as collagen, collectively promoting vascular fibrosis (Figure 2). This process also involves the recruitment of immune cells that adhere to the vascular wall and release inflammatory factors. Notably, the key inflammatory cytokines driving this abnormal cellular proliferation are highly overlapping in both diseases [3,7,21,101,102]. When this irreversible vascular remodeling causes a sustained increase in pulmonary vascular resistance, the afterload on the right ventricle escalates, ultimately triggering right ventricular decompensation and subsequent right heart failure.

Right heart failure involves multiple contributing factors. As mentioned earlier, BAS is primarily associated with disruptions in thyroid hormone secretion, ion channel dysfunction, and intracellular calcium homeostasis dysregulation. In PAH, research specifically focused on right ventricular remodeling has not received sufficient attention, and its underlying molecular mechanisms remain poorly understood. However, existing studies indicate that ion channel activity and calcium homeostasis regulate physiological processes such as electrophysiology, contractile protein activity, energy metabolism, and transcriptional regulation, and play a significant potential role in right ventricular hypertrophy and its associated dysfunction [103]. Thus, both diseases similarly rely on dysregulation of the ion channel-calcium homeostasis axis as a molecular hub, driving right ventricular hypertrophy and functional impairment. Research on left ventricular hypertrophy in PAH suggests that TRPC-dependent Ca^2+^ deposition promotes its development. In contrast, in BAS, this specific channel has not been established to contribute to the pathological progression of right ventricular remodeling, indicating a mechanistic difference between the two diseases. However, given the substantial structural and functional differences between the left and right ventricles, mechanisms identified in left ventricular dysfunction cannot be directly extrapolated to the right ventricle. The specific mechanisms involved require further clarification. The specific mechanisms of BAS and PAH were compared through different dimensions (Table 1).

### 5.2. Classification of PAH

#### 5.2.1. Idiopathic PAH

Idiopathic Pulmonary Arterial Hypertension (IPAH) lacks clearly identifiable causative factors and is understood to result from the interplay of multiple elements. It is diagnosed after all other known categories of PAH have been excluded.

Characteristic immune infiltration is observed in the lungs of patients with PAH, including the recruitment of CD4^+^ T cells, CD8^+^ T cells, γδ T cells, and M1 macrophages. Latent transforming growth factor-β binding protein 1 (LTBP1) has been shown to be closely associated with immune cell infiltration and contributes to dysregulated TGF-β signaling [110]. In addition, experimental studies based on rat models have demonstrated the presence of endothelial metabolic reprogramming in PAH. This process is accompanied by metabolic dysregulation and aberrant expression of caveolin-1 (Cav1), leading to sustained activation of HIF-1α. Consequently, both endothelial cells and pulmonary arterial smooth muscle cells exhibit suppressed oxidative phosphorylation and enhanced glycolysis, along with alterations in ion channel function [111]. In advanced stages of human PAH, progression to right heart failure results in hepatic congestion, which in turn activates the Piezo1–HIF-1α–IL-6 signaling axis. This pathway promotes hepatocellular metabolic reprogramming, central venous fibrosis, and inflammatory responses [112].

Current evidence suggests that among environmental influences, exposure to low temperatures can trigger pulmonary inflammation, promote pulmonary hypertension, and induce right ventricular hypertrophy. Sustained cold exposure exacerbates pulmonary hypertension by upregulating the expression of microRNAs (miR-146a-5p, miR-155-5p) and pro-inflammatory cytokines (TNF-α, IL-1β, IL-6) [113,114]. In high-altitude regions, healthy newborns commonly exhibit transiently low arterial oxygen saturation during the initial weeks or months of life, which typically resolves spontaneously [115]. However, whether this early hypoxic exposure predisposes these individuals to a higher lifetime risk of developing PAH compared to populations at low altitude remains an open question requiring further study. Nutritionally, deficiencies in vitamin D and iron are most frequently implicated [5,116], Insufficiencies of vitamins E, C, B12, K1, and the trace element selenium have also been associated with PAH, although the precise mechanistic links remain undefined. These deficiencies may act synergistically with other risk factors to worsen disease progression [117,118]. Dietary modulation studies in murine models indicate that a high-protein intake can attenuate inflammatory responses, reduce cardiac fibrosis, and limit skeletal muscle atrophy [119]. Selenium deficiency induces vascular remodeling by enhancing proline synthesis via *SELENBP1*, leading to fibroblast overactivation and excessive collagen deposition [120]. Furthermore, intermittent fasting has been shown to improve gut microbiota composition and cellular metabolism in mice, leading to enhanced right ventricular function [121]. In other metabolic pathways, increased arginase activity depletes arginine, thereby reducing nitric oxide (NO) production—a key vasodilatory pathway [122], Notably, while dietary arginine supplementation reduces the incidence of BAS in broilers, analogous nutritional therapeutics have not been established in human PAH. Importantly, IPAH shares several key predisposing factors with BAS, including nutritional imbalances, chronic hypoxia, and cold exposure, highlighting potential common pathways in disease modulation across species.

#### 5.2.2. Heritable PAH

In heritable PAH, a common genetic variant involves mutations in the gene encoding BMPR2, a receptor belonging to the transforming growth factor-beta (TGF-β) superfamily [123]. These mutations can lead to reduced vasodilation and abnormal proliferation of smooth muscle cells, resulting in elevated pulmonary arterial pressure. In broilers, however, mutations detected in the BMPR2 gene in breeds prone to ascites do not alter the protein sequence and may not be correlated with the occurrence of ascites [124], indicating a difference from human PAH. It should be noted, however, that the broiler strains examined in these studies were limited, and the accuracy of these findings warrants further validation. Abnormal mitochondrial fission can drive epigenetic metabolic reprogramming, contributing to right ventricular fibrosis [125]. Additionally, insufficient oxygen supply during early fetal development may induce transgenerational epigenetic changes, promoting the Warburg effect (a glycolytic shift) and mitochondrial fission [126], thereby increasing the risk of PAH later in life. While no studies have specifically examined whether site-specific mitochondrial defects in BAS are epigenetically regulated, both conditions share the common feature of energy metabolism disruption due to mitochondrial dysfunction, leading to pathological manifestations. Furthermore, PAH exhibits a gender disparity, with a significantly higher incidence in females compared to males [127], This pattern aligns with observations in BAS, although the underlying causes remain unclear. In BAS, most genes subject to adaptive mutations are associated with human cardiovascular diseases, and some of these genes are linked to gender-biased disease incidence.

#### 5.2.3. Drug- and Toxin-Induced PAH

Drug- or toxin-induced PAH is relatively rare, accounting for only 10.5% of formally reported cases. Anorexigens, such as aminorex, fenfluramine, benfluorex, phenylpropanolamine, and dexfenfluramine, were the first drugs clearly identified to induce PAH [128]. Acting as indirect serotonergic agonists and serotonin transporter substrates, these drugs elevate serotonin levels. Serotonin exerts its effects by targeting pulmonary artery smooth muscle cells and fibroblasts, and notably by upregulating the expression of the estrogen-metabolizing enzyme cytochrome P450 1B1 (CYP1B1) in smooth muscle cells. This cascade promotes pulmonary vasoconstriction and remodeling, thereby inducing pulmonary arterial hypertension and gave rise to the “serotonin hypothesis of pulmonary hypertension” [129,130,131]. Moreover, individuals with genetic susceptibility may have an increased risk of developing severe PAH when using fenfluramine [132,133]. Studies have shown that fenfluramine enhances serotonin release in Red-feather chickens, attenuating the effects of forced immobility [134], while serotonin promotes pulmonary vasoconstriction and contributes to the development of idiopathic PAH in susceptible broilers [135]. However, fenfluramine administration in drug trials did not induce PAH symptoms, which may be attributed to the short duration of the trials and differences in breed susceptibility. Further research is needed to confirm. Benfluorex, a benzoate ester with structural and pharmacological similarities to dexfenfluramine and fenfluramine, is another drug clearly associated with PAH, but its precise mechanism remains unclear [136]. Although these drugs are now prohibited from clinical use, they still hold scientific merit in medical research.

Dasatinib, a therapeutic agent for leukemia, can induce pulmonary arterial hypertension and may also cause pleural and pericardial effusions. However, treatment with dasatinib does not increase the risk of developing PAH, and symptoms are reversible in most patients upon drug discontinuation [137,138,139]. In chicken DT40B cells, dasatinib reduces hydrogen peroxide-induced phosphorylation of JNK and ERK, thereby inhibiting the activation of the downstream Syk pathway—a conserved tyrosine kinase localized at the interface between the respiratory chain and the mitochondrial intermembrane space. This disruption impairs the respiratory chain and leads to mitochondrial dysfunction, a mechanism that has been shown to be conserved in humans and mice [140].

In addition, certain drugs have been found to exacerbate several biological processes involved in the development of PAH, including mitochondrial dysfunction, oxidative stress, dysregulated cell proliferation, and disruption of intracellular ion homeostasis. This suggests that these drugs may play a regulatory role in the pathogenesis of PAH. For example, amphetamine has been shown to enhance mitochondrial oxidative phosphorylation while impairing electron transport, increasing mitochondrial reactive oxygen species (ROS) production, activating caspase-3, and inducing DNA damage [141]. Cocaine can potentiate HIV infection, exacerbate endothelial dysfunction, promote the proliferation of pulmonary smooth muscle cells, and thereby accelerate the progression of pulmonary vascular lesions [142]. During embryonic development in chickens, cocaine induces vasoconstriction and reduces fetal oxygenation, an effect that is further intensified by sodium salicylate, leading to hemorrhage [143,144,145].

#### 5.2.4. PAH Associated with Specific Conditions

PAH also occurs as a complication of other diseases, such as connective tissue diseases, portal hypertension, congenital heart disease, and schistosomiasis. In Western countries, PAH associated with connective tissue diseases is second only to idiopathic pulmonary arterial hypertension in prevalence [146], primarily driven by systemic sclerosis, whereas in Asian countries it is mainly caused by systemic lupus erythematosus (SLE) and mixed connective tissue disease [147].

SLE-associated PAH is characterized by pronounced immune activation, including enhanced IFN and TNF signaling, upregulation of endothelial adhesion molecules, and abnormal proliferation of smooth muscle cells. Notably, an inflammatory subtype of PAH exhibits a dual phenotype of concurrent immune activation and vascular remodeling [148]. Hypoxia further exacerbates disease progression through upregulation of HIF-1α, which in turn promotes vascular endothelial growth factor (VEGF) expression. This signaling axis drives proliferation of both endothelial cells and smooth muscle cells, leading to abnormal angiogenesis, increased vasoconstriction, and vascular remodeling. Importantly, the combined assessment of HIF-1α and VEGF has been proposed as a highly sensitive diagnostic biomarker for PAH. Notably, HIF-1α also represents a key target gene in broilers affected by ascites syndrome (BAS), suggesting potential mechanistic parallels between BAS and human PAH [149].

These conditions are all characterized by immune dysregulation leading to pulmonary vascular remodeling. Chickens with systemic sclerosis serve as a spontaneous autoimmune disease model for human systemic sclerosis and exhibit vascular injury pathology—a feature lacking in other animal models [150]. Furthermore, broiler chickens subjected to hypoxia-induced PAH show elevated expression of connective tissue growth factor [151].

In patients with advanced liver disease, approximately 5–6% develop portopulmonary hypertension (PoPH), which accounts for 5–15% of all PAH cases. This condition is characterized by pulmonary vasoconstriction and vascular remodeling, and is associated with a significantly lower survival rate compared to IPAH [152,153,154], Current research indicates that portal hypertension leads to splanchnic vasodilation and the formation of portosystemic shunts. As a result, a substantial volume of blood bypasses hepatic metabolism and enters the pulmonary circulation directly, creating a hyperdynamic circulatory state. Concurrently, an imbalance in vasoactive mediators—including pulmonary vasodilators such as nitric oxide and prostacyclin, and vasoconstrictors such as endothelin-1, thromboxane A2, and serotonin—drives pulmonary vascular remodeling [155,156]. Furthermore, studies have shown that broilers with pulmonary aspergillosis can develop right heart failure, followed by portal hypertension and ascites, ultimately leading to pulmonary hypertension. However, it remains unclear whether the underlying mechanisms are consistent with those of human PoPH [157].

Congenital heart disease represents a significant etiological factor in PAH, accounting for up to 30% of adult PAH cases and 75% of pediatric PAH cases (PAH-CHD). However, the underlying mechanisms remain incompletely elucidated due to the complexity and broad spectrum of cardiac anatomy and physiology, as well as numerous adaptive processes that are not yet fully understood [158,159]. Among adult PAH patients, 14.9% may develop secondary hyperparathyroidism, which is particularly common in those with right heart involvement [160]. Congenital heart disease is also associated with specific dysregulation of miRNAs and aberrant expression of extracellular matrix proteins, processes linked to cardiovascular remodeling, cell death, and right ventricular dysfunction [161,162]. Studies in chicken embryos have shown that latent hypoxia can predispose individuals to congenital heart disease, which may further increase susceptibility to cardiovascular disease in adulthood [163].

Approximately 5–10% of patients with hepatosplenic schistosomiasis develop schistosomiasis-associated pulmonary arterial hypertension (Sch-PAH), which ranks among the most prevalent forms of PAH worldwide. The pulmonary vascular pathology in Sch-PAH resembles that of IPAH, characterized by plexogenic arteriopathy and increased perivascular infiltration of T-lymphocytes and mast cells [164,165]. Current studies based on murine models suggest that pulmonary vascular injury may result from the embolization of parasite eggs into the pulmonary circulation via portosystemic shunts, eliciting a localized type 2 inflammatory response and vascular remodeling that triggers a self-sustaining, antigen-independent pathology [166]. Although no established therapeutic protocol exists specifically for Sch-PAH, drugs used in the treatment of IPAH—such as phosphodiesterase-5 inhibitors and endothelin receptor antagonists—have shown efficacy in ameliorating PAH symptoms in this population [166,167]. To date, no relevant studies on this disease have been reported in avian models.

#### 5.2.5. PVOD/PCH and PPHN

Pulmonary veno-occlusive disease/pulmonary capillary hemangiomatosis (PVOD/PCH) is a rare form of pulmonary arterial hypertension characterized by predominant involvement of the venular and capillary beds [168]. Initially classified under idiopathic PAH, PVOD/PCH exhibits distinct histopathological features. While idiopathic or heritable PAH is marked by significant remodeling of small precapillary pulmonary arteries with typical plexiform and/or thrombotic lesions, PVOD preferentially affects the postcapillary pulmonary venules [169]. Both genetic and environmental factors are associated with PVOD/PCH. Biallelic mutations in the *EIF2AK4* gene underlie the heritable form, whereas occupational exposure to organic solvents, chemotherapy, and tobacco smoke are major environmental risk factors, with the disease showing a higher prevalence in males [169,170]. In chicken embryos, exposure to cigarette smoke condensate can cause vascular misalignment, hemorrhage, and focal necrosis, potentially inducing embryonic vascular remodeling and predisposing to various diseases [171]. Furthermore, sustained inhalation of sidestream smoke equivalent to one cigarette by adult roosters has been shown to promote the formation of atherosclerotic plaques [172]. However, the vascular pathology observed in adult chickens exposed to secondhand smoke differs from the histopathological features of human PVOD/PCH.

Persistent Pulmonary Hypertension of the Newborn (PPHN) is defined as the failure to undergo the normal transitional decrease in pulmonary vascular resistance at birth. This term refers to a broad spectrum of disease states with varying pathobiological bases, yet shares the same hemodynamic definition as pulmonary arterial hypertension in adults [173,174]. PPHN can lead to enhanced peripheral vasodilation, impaired left ventricular function, and reduced preload, consequently resulting in systemic hypotension. In neonates, poor perfusion and hypoxemia may cause end-organ damage [175], making timely control of disease progression critical. Potential risk factors include maternal active or passive smoking during pregnancy, cesarean delivery, meconium aspiration, pulmonary hypoplasia, and polycythemia [176,177,178], The etiological influences are complex, and the diagnostic presentation varies significantly depending on the underlying cause. Due to these heterogeneous characteristics, mechanistic research on PPHN remains challenging. In a study using chicken embryos chronically exposed to cigarette smoke extract (CSE), it was found that CSE impairs endothelium-dependent relaxation in pulmonary arteries [179].

## 6. Limitations of BAS as a Model of Spontaneous PAH

The observed phenotypic and mechanistic overlap between BAS and PAH provides a rationale for proposing BAS as a potential spontaneous model of PAH. However, as an oviparous species, chickens exhibit fundamental differences from mammals in respiratory anatomy, physiology, developmental biology, erythrocyte structure, and lung-specific immune responses (specific differences are shown in Table 2). These interspecies differences impose important constraints on the translational applicability of BAS to human PAH.

From a hemodynamic perspective, the normal resting mean pulmonary arterial pressure (mPAP) in humans is approximately 14 ± 3 mmHg, with ≥20 mmHg defined as the diagnostic threshold for pulmonary hypertension according to the 2022 ESC/ERS guidelines. In contrast, birds such as chickens—adapted for flight and highly efficient gas exchange—display substantially higher baseline mPAP values, typically ranging from 20 to 30 mmHg or higher. This elevated physiological baseline complicates interpretation, as “mild” increases in chickens may not correspond to pathological changes in humans.

Marked structural differences in the pulmonary vasculature further limit direct comparisons. The avian lung consists of a rigid, tubular parabronchial system lacking the dense alveolar–capillary network characteristic of mammals. Additionally, differences in pulmonary arterial branching patterns and the distribution of vascular muscularization lead to distinct pressure–flow relationships, such that parameters including pulmonary vascular resistance and compliance cannot be directly extrapolated across species. Hematological differences also exist: avian erythrocytes are nucleated, and platelet function and coagulation cascades differ substantially from those in humans. Notably, even under conditions of markedly elevated mPAP, chickens rarely develop in situ thrombosis in small- to medium-sized pulmonary arteries—a hallmark of human PH—thereby limiting the suitability of BAS for modeling thrombotic PH.

Species-specific differences in the immune system represent an additional major limitation. Variations in immune cell subsets are evident: chickens lack a clearly defined Th17 equivalent, and although IL-17–producing cells are present, their phenotype and regulation by the transcription factor *RORγt* differ from those in humans. Regulatory T cells (Tregs) are present, but their suppressive capacity and surface marker expression (e.g., CD25) differ significantly, and canonical markers such as FoxP3 are less reliable for identification. Furthermore, chickens lack key immune cell populations found in humans, including plasmacytoid dendritic cells (pDCs), the principal source of type I interferons, and specific natural killer (NK) cell subsets such as *CD*56^bright^ cells.

Differences in surface markers and receptor structures further complicate immunological analyses. Many critical human immune molecules (e.g., CD20, certain epitopes of CD4, and the extracellular domain of CTLA-4) exhibit low homology or structural divergence in chickens, preventing recognition by human-specific monoclonal antibodies. This severely restricts the use of flow cytometry and immunohistochemistry for high-resolution immune phenotyping and functional assays in avian models.

In addition, immunoglobulin repertoires differ substantially between species. Chickens possess only three antibody isotypes (IgY, IgM, and IgA). Although IgY is functionally analogous to human IgG, it lacks a hinge region and does not include subclasses comparable to human IgG1 or IgG3, which mediate distinct effector functions. As a result, antibody-dependent cellular cytotoxicity (ADCC) and complement-dependent cytotoxicity (CDC) cannot be accurately recapitulated in chickens.

Collectively, these differences limit the ability of BAS to fulfill key requirements for studying immune infiltration in PAH, particularly in terms of precise immune cell subset identification, multi-marker colocalization, and validation of antibody-mediated effector mechanisms.

## 7. Discussion

Current research indicates a high degree of overlap between the pathogenic mechanisms and pathological progression of BAS and PAH, supporting the use of broilers affected by BAS as a promising spontaneous avian disease model in related PAH studies, with substantial potential for human disease research. However, given inherent interspecies limitations and the functional uncertainty of overlapping features, further systematic validation is still required at the hemodynamic, molecular, and pharmacological levels. Nevertheless, the available findings collectively indicate that BAS may represent a promising and potentially valuable model for human disease research, particularly in the context of PAH. PAH, a subclass of PH, is characterized by pulmonary vasoconstriction, endothelial injury, smooth muscle proliferation, fibrosis, increased pulmonary vascular resistance, and right heart failure. As a complex human cardiovascular disorder, its etiology is multifactorial and its developmental process is difficult to replicate. Although multiple animal models are currently employed in combination to investigate its molecular mechanisms, existing models do not fully recapitulate the complete pathological course, initial etiology, or early pathogenesis of PAH, making a comprehensive analysis of its disease mechanisms challenging. Notably, there is overlap in the pathogenic mechanisms between BAS and PAH, including pulmonary vascular smooth muscle cell proliferation, endothelial dysfunction, activation of the inflammation–fibrosis axis, elevated pulmonary vascular resistance, and right heart failure, indicating overlapping pathogenic pathways. Furthermore, studies using chicken embryos and chickens under various PAH-inducing conditions have shown certain mechanism similarities. Utilizing BAS-affected chickens as a spontaneous animal model for PAH can make up for the advantages not available in other model systems.

From a biological perspective, the chicken genome exhibits approximately 75.3% homology with the human genome on average. Research in chickens has contributed fundamental biological concepts of B and T lymphocytes, mechanisms of immunological tolerance induction, and insights into graft-versus-host disease, thereby supporting advances in human immunology. Furthermore, the chicken serves as an early and established model for human cardiovascular system development, with its external embryonic development and easy accessibility providing significant advantages for studying cardiovascular formation and function. These attributes collectively support its utility in analyzing the pathological progression, gene regulation, and immune-related molecular mechanisms underlying PAH. Chickens also offer practical advantages such as rapid development, low rearing costs, high genetic diversity, and distinct physiological traits, making them suitable for large-scale mechanistic investigation and experimental validation. Secondly, BAS occurs spontaneously, which better recapitulates the natural course of PAH. Its short disease duration and rapid progression facilitate the study of early pathogenic mechanisms and the evaluation of potential interventions. Given the etiological factors of BAS, it represents an ideal model for investigating environmental and nutritional influences, particularly suited for exploring how high altitude, low temperature, high-energy diets, and high sodium intake contribute to the induction of human PAH. Moreover, the pathogenic mechanisms of PAH induced by various etiologies may share correlations with those observed in chickens under similar stressors. Intervention experiments can also be designed based on specific causative factors to establish animal models for in-depth analysis of disease mechanisms. Among the currently established animal models of pulmonary arterial hypertension (PAH), the chronic hypoxia model is simple to establish and highly reproducible; however, it induces only mild and reversible vascular lesions. The Sugen/hypoxia (Su/Hx) model can generate plexiform and occlusive vascular remodeling and is considered the closest approximation to severe human PAH, although its phenotype is strongly influenced by animal strain and sex. The monocrotaline (MCT) model is rapid and cost-effective and induces pronounced pulmonary hypertension and right ventricular hypertrophy; nevertheless, its systemic toxicity and pathological features differ substantially from those of human PAH. The schistosomiasis-associated model is suitable for studying infection-related pulmonary hypertension, but marked species- and sex-dependent variability exists. The bleomycin model mimics pulmonary fibrosis-associated PH, although the PH phenotype is relatively mild. Mitomycin C (MMC)-induced models are regarded as ideal for pulmonary veno-occlusive disease (PVOD) research, yet they remain infrequently used. Surgical models are valuable for investigating high-flow pulmonary hypertension and right heart failure, but they are technically demanding and associated with high mortality. Gene-editing models are well suited for exploring the mechanisms underlying heritable PAH; however, their construction is complex and they generally exhibit only mild phenotypes. Multifactorial combined models more closely resemble severe PH but are accompanied by increased animal mortality rates [192].

As a classical animal model of PAH, the Fawn-Hooded rat (FHR) recapitulates the natural development of idiopathic and heritable PAH, involving mitochondrial dysfunction, Kv channel abnormalities, and dysregulation of specific signaling pathways. Importantly, its pathological features—particularly metabolic reprogramming—closely resemble those observed in human PAH [193]. However, the genetic defects in FHRs affect not only the pulmonary circulation but also the systemic vasculature, frequently leading to systemic hypertension, renal dysfunction, and alcohol preference, thereby confounding studies focused specifically on pulmonary vascular pathology [194]. In addition, FHRs are born with a platelet storage pool deficiency that is not directly causally related to PH but may introduce confounding variables involving coagulation and serotonin signaling [195]. Moreover, the model develops reliably only under conditions of mild chronic hypoxia, whereas spontaneous severe PH rarely occurs under normoxic sea-level conditions, making the stability and reproducibility of the model highly dependent on housing environment and experimental conditions [196]. Furthermore, because disease onset in FHRs typically occurs after approximately 20 weeks of age, this model is associated with prolonged experimental duration, increased cost, and heightened ethical concerns [193]. Compared with the limitations of these existing animal models, BAS may serve as a complementary model for PAH research.

Using chickens as an animal model for PAH also presents certain limitations. Beyond the interspecies differences discussed above, chickens and humans differ fundamentally in baseline hemodynamics, regulatory mechanisms, pathological responses, and disease temporal dynamics. In addition, the genetic background of broiler lines susceptible to BAS has not been systematically characterized, and their genetic stability and experimental reproducibility remain to be further validated. A major limitation lies in disease progression. BAS typically develops rapidly within 1–2 weeks. Its primary driver is the intensive growth selection in modern broiler breeding, which results in extremely high metabolic demand and increased oxygen consumption. When cardiopulmonary compensatory capacity is exceeded, pulmonary arterial pressure rises sharply, leading to right ventricular failure and ascites. The disease course is therefore acute or subacute in nature. In contrast, PAH—except for certain acute forms such as pulmonary embolism-associated cases—is predominantly a chronic and progressive disease. It evolves over years or even decades, progressing from endothelial dysfunction and medial hypertrophy to complex plexiform lesions, with clinical symptoms often appearing only in advanced stages. Due to its short disease course, the BAS model cannot recapitulate the long-term cumulative vascular remodeling observed in human PAH, nor can it capture the gradual transition between adaptive compensation and decompensation.

Regarding etiological factors, BAS is highly dependent on both environmental conditions and genetic selection. Hypoxia, high stocking density, low temperature, and energy-rich diets can induce or exacerbate disease development. It is essentially a production-related metabolic disorder reflecting the imbalance between rapid growth and cardiopulmonary reserve. In contrast, PAH is a multifactorial disease with diverse etiologies. Moreover, direct comparative clinical datasets between BAS and human PAH remain limited, further restricting formal cross-species validation.

In summary, chickens are not suitable as a standalone model for quantitative simulation of pulmonary hypertension pressure thresholds or for evaluating prognostic indicators of right ventricular failure. Rather, they are more appropriately considered complementary or screening models, particularly for studying rapid hypoxia adaptation. Interpretation of hemodynamic parameters must remain strictly confined to species-specific physiological contexts and should not be directly extrapolated to human diagnostic or therapeutic decisions. Nevertheless, existing evidence suggests that BAS shares notable face validity and construct validity with PAH, supporting its potential as a spontaneous animal model. This provides a novel perspective for the development and refinement of experimental models in PAH research.

## Figures and Tables

**Table 1:** Comparison of different PAH types and BAS across various dimension.

Comparison Dimension	Broiler Ascites Syndrome (AS)	Human Idiopathic PAH	Human Heritable PAH	Human Drug/Toxin-Induced PAH	Human Comorbidity-Associated PAH
Immune Infiltrating Cells	Macrophages and neutrophils predominate, with a small number of lymphocytes	CD4+ T cells, CD8+ T cells, γδ T cells, M1 macrophage recruitment, monocytes, and B cells	No specific immune cell subsets identified (primarily interstitial inflammation)	Macrophages and T cells predominate	T/B cells, mast cells, and monocytes
Key Inflammatory Mediators	IL-6, TNF-α, ET-1, ROS, IL-8, IL-1β, IL-18, IL-7	Elevated LTBP1, abnormal TGF-β signaling, and increased IL-1β	Mild inflammation, with TGF-β/BMP pathway imbalance	Elevated serum serotonin, IL-6, and TNF-α	Increased IFN-γ, TNF-α, and endothelial adhesion molecules
Core Regulatory Pathways	HIF-1α/VEGF, IL-6/STAT3, TGF-β, PGC-1α, mitochondrial dysfunction, NO/cGMP imbalance, DNA repair, G protein-PLC-IP3, Jak-STAT	Abnormal Cav1, sustained HIF-1α activation, Piezo1-HIF-1α-IL-6 axis, glycolysis, and activin-Smad2/3 pathway	BMPR2/Smad defects, mitochondrial fission, Warburg effect, NF-κB/STAT3/HIF-2α, and activin-Smad2/3 pathway	Serotonin pathway, CYP1B1, JNK/ERK, Syk, and activin-Smad2/3 pathway	HIF-1α/VEGF, TGF-β, adhesion molecule signaling, and activin-Smad2/3 pathway
Key Effector Cells	Endothelial cells, smooth muscle cells, fibroblasts, and right ventricular cardiomyocytes	Endothelial metabolic reprogramming, smooth muscle proliferation, and right ventricular cardiomyocytes	Fibroblasts, smooth muscle cells, endothelial cells, and right ventricular cardiomyocytes	Smooth muscle cells, fibroblasts, endothelial cells, and right ventricular cardiomyocytes	Endothelial cells, smooth muscle cells, and right ventricular cardiomyocytes
Metabolic and Ionic Features	Glycolysis, oxidative phosphorylation, ion channel abnormalities, thyroid hormone dysfunction	Metabolic dysregulation, glycolysis ↑, oxidative phosphorylation ↓	Mitochondrial impairment and energy metabolism disorders	Mitochondrial ROS ↑ and oxidative stress	Hypoxic metabolism and VEGF upregulation
Nutritional/Environmental Triggers	Low temperature, hypoxia, high-nutrition diets, selenium/vitamin deficiencies	Low temperature, hypoxia, vitamin D/iron/selenium deficiencies	Genetically driven, with nutritional factors as modifiers	Drug/toxin exposure	Underlying comorbidities, hypoxia, and nutritional imbalance
Nutritional/Additive Interventions	Arginine, selenium, vitamin E, vitamin C, and high-protein diets show significant efficacy	Vitamin supplementation, high-protein diets, and intermittent fasting may improve outcomes	Adjunctive only; cannot correct genetic defects	Nutritional interventions are ineffective	Control of underlying diseases, nutritional support
Major Pathogenic Drivers	Rapid growth, cold stress, hypoxia, and high-energy diets	Multifactorial; no clear single trigger	BMPR2 gene mutations	Fenfluramine, dasatinib, cocaine, etc.	Connective tissue diseases, portal hypertension, congenital heart disease, schistosomiasis
Sex Differences	No significant sex disparity	Higher incidence in females	Higher penetrance in females	No significant difference	Predominantly female
Ref.	[24,25,104,105,106,107,108,109]	[110,111,112,113,114,115,116,117,118,119,120,121,122]	[123,124,125,126,127]	[128,129,130,131,132,133,134,135,136,137,138,139,140,141,142,143,144,145]	[146,147,148,149,150,151,152,153,154,155,156,157,158,159,160,161,162,163,164,165,166,167]

**Table 2:** Comparison of pulmonary anatomy, respiratory physiology, and erythrocyte structure between humans and chickens.

Category	Feature	*Gallus domesticus*	Human	Ref.
Lung structure and respiratory physiology	Lung tissue color	pink	Dark red	[180,181]
	Anatomical location	Dorsal thorax	Within the thoracic cavity, flanking the mediastinum	[180,181]
	Lobation	Non-lobated	Left lung: 2 lobes; right lung: 3 lobes	[180,181]
	Volume and elasticity	Small volume, low elasticity, fixed lung parenchyma with no significant respiratory expansion/contraction	Large volume, soft and spongy, capable of active expansion and contraction during respiration	[180,181,182]
	Basic functional unit	Lack alveoli; air sacs as core structures	Possess alveoli	[180,181]
	Bronchial architecture	Interconnected tubular network forming a circular bronchial circuit with penetrating capillaries	Arborizing branching pattern terminating in alveoli	[180,181,182]
	Respiratory mode	Air sac-driven unidirectional continuous airflow	Pleural negative pressure-driven reciprocating tidal flow	[180,181]
	Gas exchange site	Capillaries	Alveoli	[180,181]
	Adaptive significance	Efficient gas exchange adapted for flight	Typical mammalian respiratory pattern	[180,181]
Red blood cell characteristics	Nucleus	Nucleated	Anucleated	[183,184]
	Cell morphology	Larger, nucleated	Biconcave disk shape	[183,184]
	Cellular deformability	Poor; adapted for flow in large-diameter vessels	High; capable of traversing microcapillaries	[183,184]
	Mitochondria	Present	Absent	[183,184]
	Energy metabolism	Aerobic respiration	Anaerobic glycolysis	[183,184]
	Hemoglobin type	HbA (α^A^_2_β^2^), HbD (α^D^_2_β^2^)	HbA (α_2_β_2_)	[183,184]
	Major regulatory molecule	Inositol pentaphosphate	2,3-Bisphosphoglycerate	[183,184]
	Thermal stability	Higher	Lower	[183,184]
	Oxygen affinity	Lower than humans	Higher than chickens	[183,184]
Lung development	Developmental origin	Foregut endoderm	Foregut endoderm	[185,186,187]
	Core regulatory conservation	Highly conserved with mammals (mouse/human): 273, 344, and 385 sequentially expressed genes shared by epithelium, mesenchyme, and endothelium, respectively; conservation depends on NKX2-1, FGFR2, TBX4/5, WNT2/2b, and Wnt, BMP, TGF-β, FGF signaling pathways	Identical to chicken (all above genes are conserved based on mouse single-cell and adult human lung atlases)	[185,186]
	Alveolar cell types	Only a single AT2-like respiratory epithelium; no functional AT1 cells	Mature AT1/AT2 alveolar cells, specifically expressing SFTA2, AGER, etc.	[186,187]
	Lung bud emergence	Embryonic day 3 of incubation	Gestational week 4	[188,189]
	Main branch formation	Approximately embryonic day 4 of incubation	Embryonic weeks 4–7	[182,188,190]
	Basic structural maturation	Approximately embryonic day 4 of incubation	Pseudoglandular stage (gestational weeks 5–17)	[182,188,191]
	Gas exchange zone formation	Late incubation to post-hatching (parabronchi mostly formed by post-hatching day 15)	Canalicular stage and beyond	[188,191]
	Maturation/alveolar (parabronchial) development	Parabronchial network formed embryonically (present at hatching)	Alveolar development mainly occurs from gestational week 36 to 3 years postnatally	[182,188]

## Data Availability

All required data are available in the manuscript. Any additional data can be provided upon request.

## Abbreviations

The following abbreviations are used in this manuscript:

AGTR1	angiotensin II receptor type 1
ALDH7A1	aldehyde dehydrogenase 7 family member A1
BAS	Broiler Ascites Syndrome
CaSR	calcium-sensing receptor
CDH6	cadherin 6
CPQ	carboxypeptidase Q
DHX58	DExH-box helicase 58
GGT5	gamma-glutamyl transferase 5
HIF 1α	hypoxia inducible factor 1α
IGSF1	immunoglobulin superfamily member 1
LRRTM4	leucine-rich repeat transmembrane neuronal 4
MC4R	melanocortin 4 receptor
PLEKHA7	pleckstrin homology domain containing A7
SOCS3	suppressor of cytokine signaling 3
SPAG1	sperm associated antigen 1
STAT5	signal transducer and activator of transcription 5
TGF-β	transforming growth factor-beta
THRα	thyroid hormones and their receptor
TREML2	triggering receptor expressed on myeloid cells like 2
TRPC	transient receptor potential cation channels
USP36	ubiquitin specific peptidase 36
UTS2D	urotensin 2 domain containing
